# More Than an Adipokine: The Complex Roles of Chemerin Signaling in Cancer

**DOI:** 10.3390/ijms20194778

**Published:** 2019-09-26

**Authors:** Kerry B. Goralski, Ashley E. Jackson, Brendan T. McKeown, Christopher J. Sinal

**Affiliations:** 1 College of Pharmacy, Faculty of Health, Dalhousie University, Halifax, NS B3H 4R2, Canada; Kerry.Goralski@Dal.Ca; 2 Department of Pharmacology, Faculty of Medicine, Dalhousie University, Halifax, NS B3H 4R2, Canada; a.jackson13@dal.ca (A.E.J.); Brendan.McKeown@Dal.Ca (B.T.M.); 3 Department of Pediatrics, Faculty of Medicine, Dalhousie University, Halifax, NS B3H 4R2, Canada

**Keywords:** cancer, obesity, adipokine, chemerin, chemokine-like receptor 1, G protein-coupled receptor 1, C-C chemokine receptor-like 2

## Abstract

Chemerin is widely recognized as an adipokine, with diverse biological roles in cellular differentiation and metabolism, as well as a leukocyte chemoattractant. Research investigating the role of chemerin in the obesity–cancer relationship has provided evidence both for pro- and anti-cancer effects. The tumor-promoting effects of chemerin primarily involve direct effects on migration, invasion, and metastasis as well as growth and proliferation of cancer cells. Chemerin can also promote tumor growth via the recruitment of tumor-supporting mesenchymal stromal cells and stimulation of angiogenesis pathways in endothelial cells. In contrast, the majority of evidence supports that the tumor-suppressing effects of chemerin are immune-mediated and result in a shift from immunosuppressive to immunogenic cell populations within the tumor microenvironment. Systemic chemerin and chemerin produced within the tumor microenvironment may contribute to these effects via signaling through CMKLR1 (chemerin_1_), GPR1 (chemerin_2_), and CCLR2 on target cells. As such, inhibition or activation of chemerin signaling could be beneficial as a therapeutic approach depending on the type of cancer. Additional studies are required to determine if obesity influences cancer initiation or progression through increased adipose tissue production of chemerin and/or altered chemerin processing that leads to changes in chemerin signaling in the tumor microenvironment.

## 1. Obesity and Cancer

Overweight and obesity rates have increased steadily for several decades and at present are a major global health crisis of epidemic proportions [1]. Recent estimates indicate that approximately 1.5 billion adults are overweight, while a further 600 million are obese [1,2]. While the rise of obesity prevalence has slowed in some countries, it is predicted that global rates will continue to increase with time and thereby exacerbate the health impact of this disorder [3]. Obesity is directly linked to a decline in quality of life and overall reduced life-expectancy as well as being a major risk factor for several prevalent metabolic, cardiovascular, and malignant disorders. Among these, cancer continues to be a leading cause of death worldwide that is attributable to an estimated 14 million incident cases and 8 million deaths annually [2,3]. In addition to other well-established risk factors for cancer (e.g., genetics, tobacco use, ionizing radiation, environmental exposure), obesity is now recognized as a risk factor for several malignancies [4,5]. These include cancers of the digestive and secretory systems (e.g., colon, stomach, liver, esophagus, kidney, gallbladder), female and male reproductive systems (e.g., ovary, postmenopausal breast, endometrium, prostate), and hematological systems (e.g., non-Hodgkin’s lymphoma, multiple myeloma, leukemia) [6,7,8,9]. Thus, with the increasing prevalence of obesity in our society, it is predicted that this disorder will soon surpass smoking as a leading significant preventable cause of cancer [10].

## 2. Role of Adipokines

While the linkage between obesity and cancer risk is an active area of investigation, the underlying biological mechanisms are not well understood. Moreover, many tumors develop in an adipocyte-rich environment. For example, adipocytes are a major cellular component of the mammary fat pad, and recent evidence indicates that these cells have dynamic interactions with cancer cells to modulate tumor growth and metastases [11,12]. Thus, local and ectopic fat depots may have an impact on cancer development that is not reflected or predicted by overall fat mass. The local and systemic alterations in physiology that are associated with obesity have the potential to impact cancer in many respects through direct effects on cancerous cells or indirect effects on the tumor microenvironment or immune function. As such, obesity can impact tumor initiation, metabolic reprogramming, angiogenesis, progression, and response to therapy variously.

Obesity is characterized not only by a generalized expansion of adipose, but also the development of a progressive metabolic and endocrine dysfunction characterized by profound alterations in the production of several factors including lipids, hormones, pro-inflammatory cytokines, and a suite of adipose derived-signaling molecules termed adipokines [13,14]. Adipokines are a heterogeneous group of peptides, mainly produced by adipose tissue, that fulfill critical regulatory roles in energy homeostasis and metabolic health [15,16]. Obesity-related alterations in the amounts and/or spectrum of adipokine release have been linked to metabolic disorders such as hyperlipidemia and type 2 diabetes and are increasingly recognized as a key factor linking obesity with cancer. For example, adiponectin is an adipokine with established pleiotropic roles in regulating insulin-sensitivity as well as lipid and glucose homeostasis [4]. Circulating levels of adiponectin are inversely correlated with adiposity and this is believed to contribute to the increased risk for obesity-related comorbidities such as type 2 diabetes and metabolic syndrome [4]. Lower levels of this adipokine have also been linked to an increased risk for several types of cancer [17,18]. In contrast to adiponectin, circulating levels of the adipokine leptin increase in proportion to fat mass. While different epidemiological studies have offered conflicting results regarding the impact of leptin on general cancer risk, a recent meta-analysis of 23 studies reported a positive association with breast cancer risk [19]. Moreover, overexpression of the receptor for leptin has been found in breast cancer and in particular for higher-grade tumors associated with metastasis and poor clinical prognosis [20,21,22,23].

## 3. Chemerin

Chemerin is a multifunctional secreted protein with established roles in energy metabolism, immune function, and fundamental cell processes such as differentiation, proliferation, and chemotaxis [24,25]. Consistent with its role as an adipokine, evidence from clinical and animal studies have firmly established that secretion and circulating levels of chemerin increase with adiposity and decline after bariatric surgery, diet, and exercise-based weight loss [26,27,28,29,30,31,32,33,34,35]. In addition to adipose tissue, chemerin is highly expressed in many other human tissues including the adrenals, liver, female reproductive organs, mammary tissue, and lung (Data Source: GTEx Analysis Release V7 (dbGaP, Accession phs000424.v7.p2, accessed on 29 July 2019)) as well as cell types such as intestinal epithelial cells, platelets, keratinocytes, synovial fibroblasts, and vascular endothelial cells [36,37,38,39,40]. Therefore, when assessing a role for this adipokine in cancer, the impact of chemerin produced locally within the affected tissue and/or tumor microenvironment must be considered in addition to systemic levels of circulating chemerin. 

Chemerin is synthesized as pre-prochemerin, which requires N-terminal cleavage of a 19-amino acid signaling domain prior to its secretion as a 163-amino acid precursor (prochemerin) [37,41,42,43,44]. Subsequently, prochemerin undergoes extracellular proteolytic processing at the C-terminus exposing the active region and forming active chemerin [37,41,44]. In humans, prochemerin is processed to at least three active products; chemerin156, chemerin157, and chemerin158, all of which have been detected in biological fluids, including plasma and serum [42,45,46]. Further proteolytic events cleave active chemerin isoforms to shorter inactive or low activity proteins [35,47]. Chemerin is the endogenous ligand for two known cognate signaling receptors, chemokine-like receptor 1 (CMKLR1) and G protein-coupled receptor 1 (GPR1); herein these are referred to as chemerin receptor 1 (chemerin_1_) and chemerin receptor 2 (chemerin_2_) as established by the International Union of Basic and Clinical Pharmacology Committee on Receptor Nomenclature [48]. A third chemerin receptor, C-C chemokine receptor-like 2 (CCRL2), exhibits limited homology with chemerin_1_ and chemerin_2_ and is most closely related to the atypical chemokine receptor family [48]. Rather than directly mediating chemerin signaling, CCRL2 is thought to function as a chemerin membrane anchoring protein that increases local chemerin concentrations and presents the ligand to chemerin_1_ or chemerin_2_ expressing cells. [49,50] Depending upon the site of proteolytic cleavage and interaction with either of chemerin_1_ or chemerin_2_, the magnitude and nature of the biological effects of chemerin can vary dramatically (e.g., pro- versus anti-inflammatory) [24]. Chemerin has been shown to mediate the chemoattraction of several chemerin receptor-expressing leukocyte subsets that are often present in the tumor microenvironment, including dendritic cells, natural killer cells, and macrophages [42,51,52]. Therefore, chemerin signaling may play a role in cancer immunology through these mechanisms.

Circulating chemerin levels correlate positively with adiposity, and it is generally accepted that major peripheral white adipose depots, such as subcutaneous and visceral fat, are significant contributors to systemic chemerin levels. However, recent research indicates that locally-derived chemerin, produced either by tumors or by adipocytes in close proximity to the tumor, may have auto/paracrine effects that are distinct from the hormonal influence of systemic chemerin. The aim of this review is to summarize the evidence linking chemerin, and the cognate receptors, to the risk, mechanism, and prognosis of human cancer. Please note that this review provides complementary information to the paper by Treeck et al. [53] also published in this special issue.

## 4. Esophageal and Oral Cancers

Both systemic and tumor-localized chemerin levels are associated with pro-cancer effects in esophageal and oral carcinoma. Overexpression of chemerin has been demonstrated in oral squamous cell carcinoma (OSCC), squamous cell carcinoma of the oral tongue (SCCOT), and oesophageal squamous cancer (OSC) [54,55,56]. In a study of OSCC patients, increased circulating and salivary concentrations of both chemerin and the extracellular matrix remodeling enzyme matrix metalloproteinase-9 (MMP-9) were observed compared to patients with oral pre-malignant lesions (OPLs) and controls [54]. Table 1 summarizes the serum/plasma chemerin concentrations, as well as patient demographics (subject groups, numbers, age, sex, and BMI), for this and all other studies described in the present article. Furthermore, patients with pre-malignant lesions also displayed elevated levels of chemerin and MMP-9 when compared to healthy controls [54]. Similarly, several studies have reported increased expression of chemerin in SCCOT tissues compared to adjacent non-cancerous tissues and in OSC cancer-associated myofibroblasts (CAMs) compared to adjacent tissue myofibroblasts (ATMs) [55,57]. In SCCOT, overexpression of both chemerin mRNA and protein was correlated with a number of poor clinical indicators, including lymph node infiltration, microvessel density, tumor angiogenesis, and advanced clinical stage [54,55,58]. Furthermore, chemerin expression was greater in advanced-stage SCCOT tumors and thus, was linked to a poor prognosis [55].

**Figure 1:**
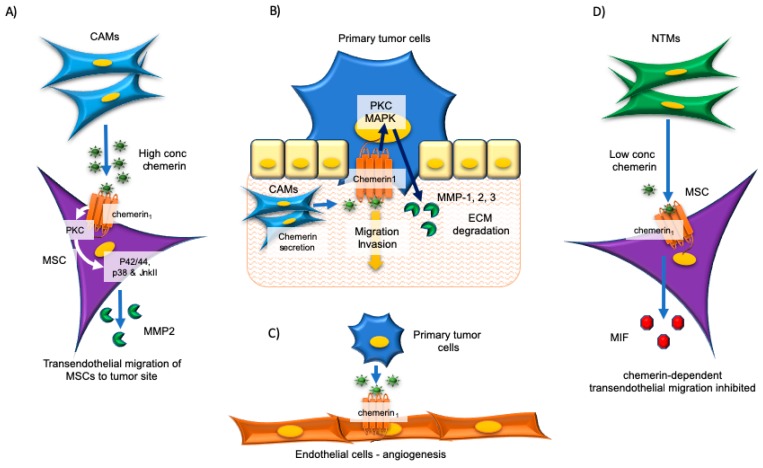
The mechanisms of tumor-promoting effects of chemerin in the esophageal carcinoma microenvironment. Chemerin is released from cancer-associated myofibroblasts (CAMs) and esophageal tumor cells and has autocrine and paracrine tumor-promoting effects in the esophageal carcinoma microenvironment. These include mediating mesenchymal stromal cell (MSC) transendothelial migration to the tumor site (**A**), tumor cell migration and invasion (**B**), and angiogenesis (**C**). In contrast, low chemerin concentrations inhibit MSC migration (**D**). ECM, extracellular matrix; MAPK, mitogen-activated protein kinase; MIF, macrophage inhibitory factor; MMP, matrix metalloproteinase; NTM, normal tissue myofibroblasts; PKC, protein kinase C.

The mechanisms by which chemerin may contribute to esophageal tumor progression are multifaceted involving multiple cell types within the tumor microenvironment (Figure 1). One mechanism involves a paracrine interaction between chemerin-secreting CAMs and chemerin_1_-expressing mesenchymal stromal cells (MSCs), leading to MSC migration into the tumor microenvironment (Figure 1, left). In in vitro transwell migration assays and transendothelial migration assays, chemerin stimulated the migration of MSCs via interactions with chemerin_1_ but not chemerin_2_ [57]. Notably, the effects on MSC migration were greater with conditioned media derived from esophageal CAMs versus that of ATMs [57]. These results were validated in an in vivo xenograft model, where BALB/c nu/nu mice injected S.C. with OE21 human esophageal carcinoma cells along with CAMs had more infiltrated MSCs than those mice injected with OE21 cells alone [57]. The in vivo homing of MSCs to the OE21 tumors was reduced by the chemerin_1_ antagonist CCX832 confirming the effect was dependent on chemerin/chemerin_1_ signaling. Evidence supported that chemerin/chemerin_1_ signaling in the MSCs is relayed via protein kinase C (PKC) and subsequent phosphorylation and activation of protein kinases p42/44, p38 and JnkII, and matrix MMP-2 secretion, which contributes to the trans-endothelial migration of MSCs, potentially contributing to cancer progression [57]. The study by Kumar et al. went a step further by providing additional evidence for a contextual pro-cancer role for chemerin in these malignancies (Figure 1, right). Unlike with high concentrations of chemerin (20 ng/mL), low concentrations of chemerin (4 ng/mL) inhibited approximately 50% of chemerin/chemerin_1_-mediated MSC migration through a 10-fold increase in the secretion of macrophage inhibitory factor (MIF) from MSCs [57]. The authors speculated that moderate levels of chemerin in normal tissue myofibroblasts (NTMs) would act to restrain MSC migration through the autoinhibitory action of MIF. However, in the tumor microenvironment, the MIF-inhibitory mechanism is released owing to higher chemerin concentrations in CAMs, increasing the capacity for recruiting MSCs to the tumor microenvironment [57]. 

A follow-up study by Kumar et al. expanded on this area of research by demonstrating paracrine interactions between chemerin-secreting CAMs and the chemerin_1_-expressing esophageal cancer cell line OE21. Conditioned media from CAMs, more so than conditioned media from ATMs and NTMs, stimulated migration and Matrigel invasion of OE21 cells, which could be partially blocked by chemerin neutralization, siRNA knockdown of chemerin or chemerin_1_, or pharmacological antagonism of chemerin_1_ with CCX832 [56]. The invasion process was mediated through PKC- mitogen-activated protein kinase (MAPK) signaling but did not require phosphoinositide 3-kinase (PI3K) and led to MMP1, 2, and 3 secretion, which may facilitate invasion through extracellular matrix degradation (Figure 1, top-centre) [56].

Chemerin has previously been shown to stimulate angiogenesis [74,75]. Thus, interactions between tumor cell-secreted chemerin and chemerin_1_-expressing endothelial cells leading to increased angiogenesis is another possible mechanism (Figure 1, bottom-centre). Supporting this idea, one study found that increased chemerin expression in SCCOT was strongly associated with increased microvessel density, an indicator of angiogenesis [55].

In the metaplasia–dysplasia–carcinoma sequence of Barrett’s esophagus (BE) to high-grade dysplasia BE and esophageal carcinoma, a significant increase in myeloid dendritic cell (mDC) and plasmacytoid dendritic cell (pDC) density was observed that coincided with increased expression of their respective chemotactic factors, macrophage inflammatory protein-3 alpha (MIP3α), and chemerin in the same regions [76]. However, the metaplasia–dysplasia–carcinoma transition was also characterized by the infiltration of immune tolerogenic IL-10^high^ and IL-12^low^ mDCs, which stimulated the differentiation of immunosuppressive T regulatory (Treg) cells from naïve CD4^+^ T cells [76]. Thus, while high tumor chemerin concentrations have an anti-tumoral effect in other cancers [52,77,78,79], these effects may be masked in the context of esophageal cancers because of an immune tolerogenic phenotype. Alternatively, chemerin could be contributing to the immune tolerogenic phenotype, but this remains to be determined experimentally. 

## 5. Colorectal and Gastric Cancer

Similar to esophageal and oral cancers, the balance of clinical evidence indicates a positive association between serum chemerin concentrations and the risk for colorectal cancer [59,60,62,63] and gastric cancer as reviewed in greater detail by Treeck et al. [53] and originally reported by Wang et al. [64] and Zhang et al. [61] (Table 1). There is considerable variability among these studies with respect to reported absolute values for serum chemerin, possibly due to methodological differences. In spite of this variability, there is a consistent finding of elevated serum chemerin in gastric and colorectal cancer patients. There is also some uncertainty as to the linkage of chemerin to colorectal cancer owing to inherent differences (e.g., age) between the patient and control groups [59]. However, other studies have reported significantly higher circulating chemerin levels after considering potential confounds such as age, sex, BMI, waist circumference, and diet. For example, after adjusting for age and sex, Eichelmann et al. [60] reported an approximate 2-fold increase in overall risk for all colorectal cancers between the highest and lowest quartile of serum chemerin concentrations. This association was strongest for colon cancer (HR = 2.27) and specifically proximal colon cancer (HR 3.97) [60]. Consistent with these findings, Alkady et al. [62] reported that using a cut off of ≥ 161.5 ng/mL, serum chemerin had 100% sensitivity and 100% specificity for the presence of colorectal cancer. Increased serum chemerin was also found to correlate with general fatigue and other cancer-related symptoms in colorectal cancer patients [63]. Moreover, progressive increases in serum chemerin have been observed in patients with advanced stages of colorectal cancer [62]. Overall, these results support a cancer and stage-specific effect on serum chemerin concentrations. These studies are also in general agreement regarding the potential for the use of chemerin as a biomarker for colorectal cancer independent of inflammatory markers such as C-reactive peptide (CRP) [59,60,62].

**Figure 2:**
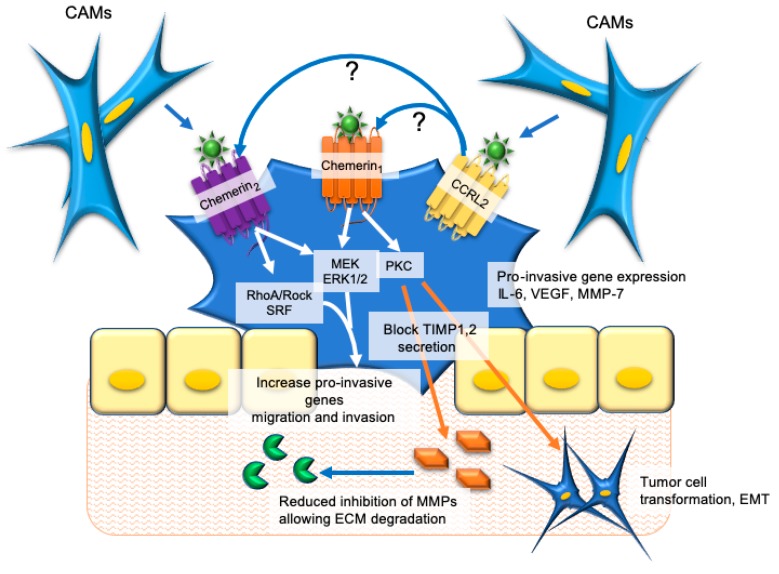
The mechanisms of tumor-promoting effects of chemerin in the gastric carcinoma microenvironment. Chemerin is released from cancer-associated myofibroblasts (CAMs) and acts on chemerin_1_ and chemerin_2_ receptors present on gastric carcinoma cells to activate several intracellular signaling pathways. Functionally this signaling leads to increased expression of pro-invasive genes, reduced secretion of tissue inhibitor of metalloproteinase 1, 2 (TIMP-1/2), and enhanced production of matrix metalloproteinases (MMPs) leading to migration and invasion of tumor cells and tumor cell transformation resembling an epithelial–to–mesenchymal transformation (EMT). It is unknown (?) how and if CCRL2-bound chemerin interacts with chemerin_1_ and chemerin_2_ to influence the tumor-promoting effects of chemerin signaling in gastric carcinoma. ECM, extracellular matrix; ERK1/2, extracellular-related kinase 1/2; IL-6, interleukin 6; MAPK, mitogen-activated protein kinase; PKC, protein kinase C; VEGF, vascular endothelial growth factor.

In this issue, Treeck et al. [53], reported that high gastric tumor expression of chemerin, chemerin_1_, and chemerin_2_ were associated with shorter overall patient survival. Consistent with these findings, the results from several in vitro studies support a tumor-promoting role of chemerin signaling in gastric cancer (Figure 2). For example, Wang et al. [64] reported that exposure of human gastric cancer AGS or MKN28 cells to recombinant human chemerin promoted invasiveness in a dose-dependent fashion in Matrigel invasion assays. This was accompanied by increased expression of a panel of “pro-invasive” genes including *vascular endothelial growth factor* (*VEGF*), *Interleukin-6* (*IL-6*), and *MMP-7* mRNA suggesting a mechanism whereby increased chemerin could increase the metastatic potential of gastric cancer cells [80,81,82,83]. When the invasion and gene expression assays were repeated in the presence of various MAPK inhibitors, the extracellular-related kinase (ERK) inhibitor UO126 most consistently blocked the effects of chemerin versus p38 and c-jUN N-terminal kinase (JNK) inhibitors, which were less effective. This suggested the effects of chemerin were primarily mediated by ERK signaling, a pathway with known involvement in the promotion of cell proliferation and migration [84]. However, there was no effect of chemerin on cell proliferation, a finding consistent with that of our research group which observed no effect of chemerin treatment on the proliferation or viability of AGS cells [85]. A new pathway for chemerin signaling through Gαi/o and RhoA/Rock was identified, which activates serum response factor regulated gene expression and chemotaxis of AGS cells [85]. It was postulated that these effects were chemerin_2_ receptor-mediated, as AGS cells were found to express *chemerin_2_* but not *chemerin_1_.* In contrast, Kumar et al. detected both chemerin_1_ and chemerin_2_ proteins using immunohistochemistry in both primary gastric cancer cells and AGS cells [86]. *Chemerin* mRNA was not expressed in AGS cells [85] nor was secreted chemerin detected in the media of cultured AGS cells [86]. However, chemerin was secreted by CAMs at concentrations sufficient to stimulate migration and morphological transformation of AGS cells [86] supporting a paracrine rather than autocrine mechanism of signaling. These effects of chemerin were inhibited by the putative chemerin receptor antagonists CCX832 and α-NETA [86]. Similarly, selective knockdown of either chemerin_1_ or chemerin_2_ resulted in inhibited migration and invasion in AGS cells, while simultaneous knockdown led to complete inhibition [86], supporting the functional signaling of chemerin_1_ and chemerin_2_ in AGS cells. These observations are consistent with clinical findings showing an increased risk for gastric cancer with increased serum chemerin. The study by Kumar et al. also uncovered the further complexity of chemerin signaling in gastric cancer by demonstrating that chemerin inhibited the secretion of tissue inhibitor of metalloproteinase 1 and 2 (TIMP -1/-2) via a PKC mediated pathway in AGS cells [86]. As TIMPs inhibit MMP activity, decreased secretion would be expected to increase metastatic and invasive potential [87]. Interestingly Treeck et al. reported that in contrast to chemerin_1_ and chemerin_2_, increased CCRL2 expression in gastric carcinoma was correlated with increased overall survival [53]. However, the mechanisms of this putative protective effect of CCRL2 remain unknown.

Expression of the non-signaling chemerin receptor, *CCRL2*, was reported to be reduced by about 2/3 in colorectal cancer patients versus disease-free controls [88]. Unlike chemerin, there was no correlation in *CCRL2* mRNA levels with colorectal cancer stage [88]. While CCRL2 expression was detectable in several colorectal cell lines (SW480, SW620, LS174T, Caco2), siRNA-mediated knockdown of *CCRL2* mRNA reduced proliferation, colony formation and migration only in LS174T cells [88]. When rat CC531 colorectal cancer cells were injected into the rat portal vein for liver colonization assays, the initial low *CCRL2* mRNA levels increased during initial colonization of the liver [88]. This suggests a linkage to tumor cell migration or invasion. Whether or not the increased CCRL2 facilitates chemerin interactions with chemerin_1_ or chemerin_2_ within this context remains to be determined.

## 6. Skin Cancer

In contrast to the aforementioned cancers, both melanoma and skin squamous cell carcinoma have been associated with decreased expression of chemerin mRNA and protein [52,89]. Available evidence suggests that this may promote skin cancer progression and tumor growth through a reduction in the recruitment of immune cells to the tumor microenvironment via chemerin-dependent mechanisms. Consistent with this, tumors with higher *chemerin* expression were associated with improved clinical outcomes in melanoma [52]. The same study found that an intratumoral injection of chemerin into a B16 transplantable mouse melanoma model resulted in reduced tumor growth [52]. The beneficial effects of chemerin in reducing melanoma progression appear to be mediated primarily through the recruitment of NK cells, and to a lesser extent, other immune effectors such as T and B cells to the tumor microenvironment [52]. In contrast, it was found that chemerin played little to no role in the activation of NK cells and had no discernible direct effects on melanoma cells [52]. 

**Figure 3:**
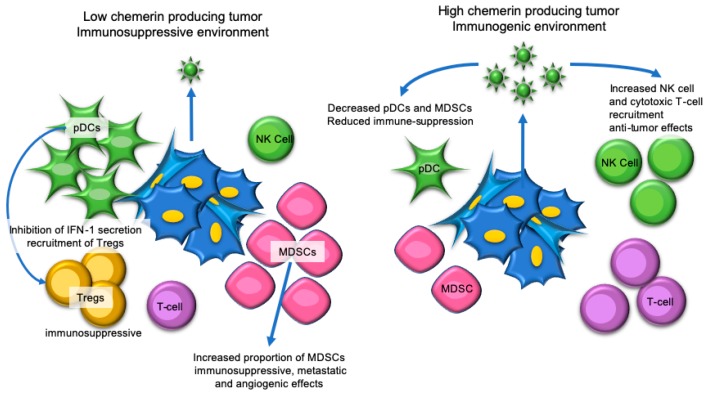
Chemerin has immune-mediated tumor-suppressive effects in melanoma. In low chemerin-producing melanoma tumors, there is an increased presence of myeloid-derived suppressor cells (MDSCs), plasmacytoid dendritic cells (pDCs), and regulatory T-cells (Tregs), which result in a tumor-promoting immunosuppressive environment. When melanomas produce higher amounts of chemerin, there is a switch to a tumor-suppressing immunogenic environment characterized by increased natural killer (NK) cell and cytotoxic T-cell infiltration and reduced infiltration of MDSCs and pDCs.

Chemerin also appears to have an important role in regulating the ratio between beneficial and harmful immune cells in the tumor microenvironment (Figure 3). As the name suggests, myeloid-derived suppressor cells (MDSCs) originate from the myeloid-lineage and contribute to tumor progression via the suppression of appropriate immune responses [90]. MDSCs exert additional pro-cancer effects through the upregulation of angiogenic and metastatic factors in the tumor microenvironment [90]. Localized chemerin expression in melanoma was associated with an increase in the ratio of immune effectors (i.e., NK cells, T cells, and dendritic cells) to MDSCs in the tumor microenvironment, ultimately enhancing anti-tumor responses [52]. Additionally, pDCs play a significant role in melanoma and have been associated with poor clinical outcomes through the development of an immunosuppressive microenvironment [91]. Normally pDCs promote anti-viral immunity, but in melanoma, the suppression of type I interferon (IFN I) production by pDCs triggers immunosuppressive mechanisms including the recruitment of Treg cells to the tumor microenvironment [91]. Localized chemerin expression in melanoma has been demonstrated to decrease the presence of pDCs in the tumor microenvironment, ultimately inhibiting immune escape mechanisms [52].

## 7. Hepatocellular Carcinoma

Similar to skin cancer, a number of studies support an anticancer role for chemerin in human hepatocellular carcinoma [77,78,92]. Collectively, these studies suggested that in certain hepatocellular carcinomas, hepatic chemerin production may be lowered, thus facilitating further advancement of the disease [77]. In contrast, increased serum chemerin concentrations have been associated with more favorable clinical characteristics, such as reduced tumor size, differentiation, and stage and indicate the potential value of chemerin as a prognostic factor for disease-free survival [78,92]. The clinical associations between chemerin signaling and hepatocellular carcinoma have been described in detail by Treeck et al. in this issue [53].

To explore the mechanisms underlying the clinical associations between chemerin signaling and hepatocellular carcinoma, Lin et al. and Li et al. utilized mouse models in which chemerin expression was manipulated in several complementary manners [77,92]. Mice injected in the left ventricle with chemerin-overexpressing portal vein tumor thrombus cells (PVTT-1-Che) only rarely developed metastatic foci, while those injected with control PVTT-1 cells consistently developed metastases at distant sites throughout the body [77]. Similarly, mice injected hepatically with PVTT-1-Che cells exhibited reduced liver tumor foci development, a 1.3-fold increase in survival (54 days versus 41 days) compared to mice injected with control PVTT-1 cells [77]. This lessening of metastasis and prolongation of survival was recapitulated by the intraventricular or intraperitoneal injection of recombinant chemerin to mice that also had an intraventricular or hepatic injection of control PVTT-1 cells [77]. Likewise, when implanted with Hepa1-6 tumor cells, chemerin knockout mice (chemerin^-/-^) developed larger liver tumors, more frequent lung metastasis and showed significantly increased mortality as compared to the wild type mice [92]. Overexpression of chemerin in Hepa1-6 cells resulted in decreased mortality and decreased liver tumor growth compared to control Hepa1-6 cells injected into wild-type mice [92].

**Figure 4:**
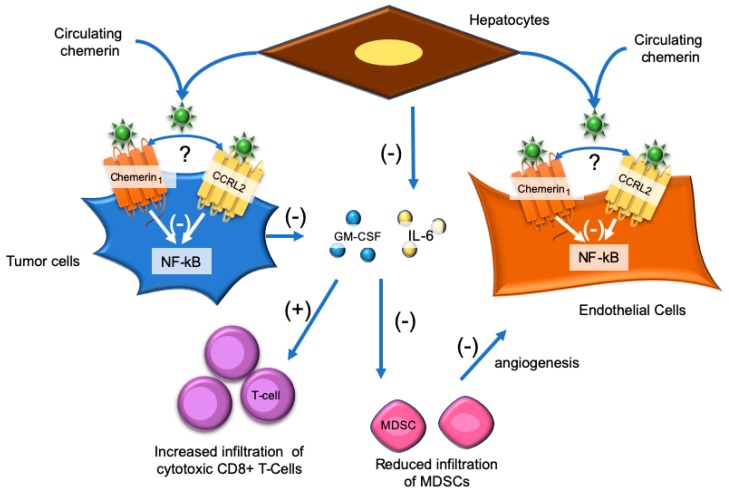
Chemerin has immune-mediated tumor-suppressive effects in hepatocellular carcinoma. Systemic or hepatocyte-secreted chemerin interacts with chemerin_1_ and CCLR2 on hepatocellular carcinoma cells and endothelial cells to inhibit nuclear factor kappa B (NF-κB) signaling. By unknown mechanisms, this leads to reduced secretion of granulocyte-macrophage colony-stimulating factor (GM-CSF) from tumor cells and IL-6 from hepatocytes. In turn, this leads to reduced tumor infiltration of immunosuppressive and pro-angiogenic myeloid-derived suppressor cells (MDSCs) and increased infiltration of cytotoxic CD8+ T-cells. It is unknown (?) how and if CCRL2-bound chemerin interacts with chemerin_1_. (−) = Reduction or suppression of a normal pathway and (+) Increase of a normal pathway.

The study by Lin et al. supports that the hepatocellular protective effects of chemerin are immune-mediated involving a shift from tumor-infiltrating immunosuppressive and angiogenesis-stimulating MSDCs to tumor-suppressing interferon γ-secreting T cells (IFNγ^+^T) (Figure 4). In support of this conclusion, Hepa1-6 tumors in chemerin^-/-^ mice displayed increased proportions of MDSCs, tumor-associated macrophages (TAMs) and decreased IFNγ-expressing T-helper CD4^+^ and cytotoxic CD8^+^ T cells compared to Hepa1-6 tumors in wild-type mice [92]. Consistent with this result, chemerin-overexpression caused a shift from MDSCs to IFN-γ^+^ T cells in the Hepa1-6 tumors [92]. An impairment but not a complete abolition of the hepatocellular carcinoma-inhibiting effect of chemerin was observed in T-cell and B-cell deficient Rag1-/- mice and CD8+ T cell-depleted mice confirming a partial role of CD8+ T cells in the antitumoral effects of chemerin [92]. There were no differences in Tregs or pDCs regardless of chemerin expression in the Hepa1-6 tumors [92]. Furthermore, there was no difference in tumor-infiltrating NK cells, which is consistent with the weak but significant positive correlation observed between human hepatocellular carcinoma chemerin expression levels and recruitment number of dendritic cells and NK cells to the tumor site [78,92]. A series of in vitro and in vivo experiments probed the cellular and molecular mechanisms of chemerin suppression of hepatocellular carcinoma progression. These studies identified that chemerin interacts with chemerin_1_ and CCLR2 to inhibit nuclear factor kappa B (NF-κB) signaling in tumor cells and endothelial cells. This leads to reduced production and secretion of the pro-tumorigenic factors, granulocyte-macrophage colony-stimulating factor (GM-CSF) from tumor cells and IL-6 from hepatocytes, which in turn suppress the numbers of tumor-infiltrating MDSCs and allows for a restoration of T-cell immunity and reduced angiogenesis in the tumor microenvironment [92]. 

**Figure 5:**
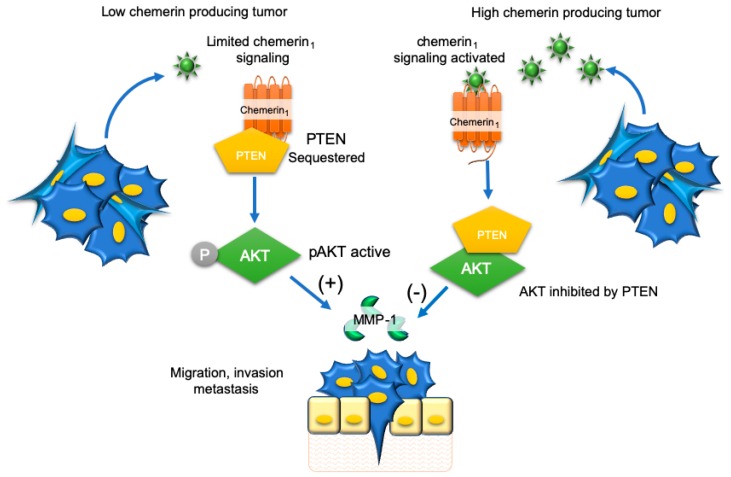
Hepatocellular carcinoma-derived chemerin inhibits tumor cell migration, invasion, and metastasis via an autocrine mechanism. When chemerin production by hepatocellular carcinoma is low (left) there is limited autocrine signaling through chemerin_1_. This results in sequestering of the tumor suppressor phosphatase and tensin homolog (PTEN) through a direct physical interaction with chemerin_1_, allowing for the activation of protein kinase B (AKT) and secretion of matrix metalloproteinase 1 (MMP-1), which is thought to facilitate migration, invasion, and metastasis. When chemerin production by hepatocellular carcinoma is high (right), chemerin_1_ signaling is activated, the chemerin_1_-PTEN complex is disrupted, allowing PTEN inhibition of AKT and blockade of migration, invasion, and metastasis of hepatocellular carcinoma. (+) = Activation, (−) inhibition.

Adding to the complexity of the actions of chemerin in this context, Li et al. demonstrated the protective effects of chemerin on the progression of hepatocellular carcinoma also involve autocrine effects of tumor cell-secreted chemerin [77]. These included a reduction in migration and invasion of multiple hepatocellular carcinoma cell lines in the presence of chemerin overexpression and a reversal of this effect with chemerin neutralizing antibodies [77]. In agreement with other studies, there was no impact of chemerin on hepatocellular carcinoma proliferation and apoptosis. Mechanistically, when chemerin concentrations were low, chemerin_1_ physically interacted with the tumor suppressor phosphatase and tensin homolog (PTEN) as demonstrated by immunoprecipitation assays (Figure 5). This led to greater ubiquitination of PTEN, lowering its activity and suppressive effects on protein kinase B (AKT) activation. On the other hand, when chemerin concentrations were increased, the interaction between chemerin_1_ and PTEN was disrupted, reducing PTEN ubiquitination and increasing its activity. In turn, AKT activation by phosphorylation was inhibited suppressing migration, invasion, and metastasis of hepatocellular carcinoma cells. Notably, in the study by Li et al., MMP-1 was increased along with AKT, whereas PTEN was decreased in metastatic foci of mice with PVTT control tumors. The opposite pattern was observed in metastatic foci of mice with PVTT-Che tumors. This suggested the antitumor effects of chemerin involve, in part, MPP-1 which is active in the promotion of tumor migration through proteolytic functions [87].

A recent study by Sun et al. reported a modest inhibitory effect of chemerin on the proliferation of SMMC7721 human hepatoma cells but not QSG7701 immortalized human hepatic cells [93]. This appeared to be a result of S-phase cell cycle block involving reductions in p53, p27, and p21 proteins. Interestingly, the mechanism involved downregulation of iron transporters and regulatory proteins, including the divalent metal transporter, transferrin, transferrin receptors 1 and 2, iron regulatory proteins 1 and 2 and ferritin-H, and ferritin-L leading to decreased cellular iron concentrations [93]. Consistent with this, iron supplementation reversed the effects of chemerin on S-phase cell cycle block and p53, p27, and p21 proteins. The results of this study contrast with others that did not observe effects of chemerin on cell proliferation or apoptosis [77,92,93]. The reason for the discrepancy is not certain, but it could relate to the different cell lines used in the three studies. Furthermore, it is worth noting that the SMMC7721 and QSG7701 cells are potentially HeLa derivatives as they have been listed as being at risk for contamination [94,95].

Not all studies support a clear relationship between chemerin and hepatocellular carcinoma. For example, Imai et al. detected no significant difference in recurrence-free survival or disease-free survival between patients classified with having low (≤ 130.5 ng/mL) and high (> 130.5 ng/mL) serum chemerin concentration [65]. Furthermore, no association was found between serum chemerin and clinical stage of hepatocellular carcinoma in this study [65]. However, a correlation was observed between serum chemerin concentration and severity of liver disease suggesting that with advancing liver disease, hepatic chemerin production decreases and may increase the risk for further advancement of hepatocellular carcinoma [65]. Haberl et al. utilized a mouse model of low methionine-choline deficient diet-induced non-alcoholic steatohepatitis (NASH) compared to NASH with dimethylnitrosamine-induced hepatocarcinoma (NASH-HCC) to evaluate the function of chemerin in NASH-HCC. Hepatic and serum chemerin, as well as ex vivo activation of chemerin_1_, did not differ in the two models. The authors concluded that tumors still develop despite high endogenous levels of serum and liver chemerin protein [96].

## 8. Adrenocortical Carcinoma 

Adrenocortical carcinoma is a rare, aggressive form of cancer with poor prognosis [97]. Through microarray analysis to identify gene signatures of potential diagnostic value, a substantial downregulation of *chemerin* expression in adrenocortical carcinoma versus benign adrenal adenomas was discovered in two independent cohorts [98,99]. These findings have been replicated in additional independent sample cohorts, which also included a comparison to control non-cancerous adrenal tissue [79,100]. *Chemerin* expression was highest in control tissue, followed by an intermediate expression in the benign adrenal adenomas and lowest in the carcinomas. A positive correlation was observed for immunohistochemical detection of the chemerin protein in paired samples, providing evidence that reduced *chemerin* expression coincides with reduced chemerin protein [79]. The mechanism of reduced *chemerin* expression in adrenocortical carcinoma appears to be through repressive hypermethylation at 5 CpG sites, which could be reversed by the DNA-methyltransferase inhibitor decitabine [79].

Despite the significantly lower *chemerin* expression, a survival analysis of four independent data sets comparing subjects with the highest (top 50%) to lowest (bottom 50%) *chemerin* expression within adrenocortical carcinoma tissue revealed no significant difference [100]. Somewhat paradoxically, serum chemerin concentrations were increased in adrenocortical carcinoma subjects versus those with benign adenoma or healthy controls and were positively associated with longer overall survival [100]. To further assess the relationship between adrenal *chemerin* expression and serum chemerin concentrations, the researchers xenografted immunodeficient scid-γ mice with H295R adrenocortical carcinoma cells with and without human *chemerin* overexpression. The tumors, with higher *chemerin* expression, had higher serum human chemerin. Based on this result, the authors rationalized that since chemerin decreases in adrenocortical tumors, the increased serum chemerin concentration must be due to chemerin secretion from tissues other than the adrenals, but the exact tissues were not identified. Adipose tissue was ruled out as a contributor to increased serum chemerin for a number of reasons, but this was not confirmed experimentally [100]. Interestingly, mice transplanted with human *chemerin*-expressing H295R tumors had higher serum concentrations of human chemerin but proportionally lower mouse serum chemerin suggesting a type of negative regulatory feedback mechanism. The overall findings led the authors to reasonably postulate that the reduction in adrenal tumor chemerin concentrations could be an immune avoidance mechanism, but increased serum chemerin may counteract this in some individuals resulting in improved anti-tumor immune responses. While not tested in this study, it represents an interesting idea for a follow-up.

**Figure 6:**
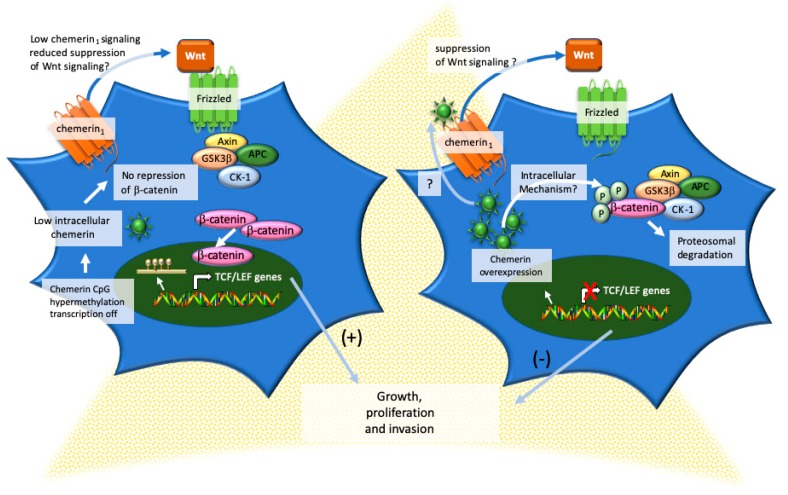
Endogenously derived chemerin mediates a tumor-suppressive effect through inhibition of Wnt/β-catenin signaling in adrenocortical carcinoma. In adrenocortical carcinoma, chemerin expression becomes suppressed due to CpG hypermethylation resulting in low intracellular chemerin concentrations. β-catenin accumulates and migrates to the nucleus where TCF/LEF genes are turned on mediating (+) cell growth, proliferation, and invasion. Based on the known feedback inhibition of chemerin_1_ on Wnt/β-catenin signaling, it is also possible that low chemerin_1_ expression could contribute to the activation of Wnt/β-catenin in adrenocortical carcinoma cells. When tumor chemerin production is increased, by unknown (?) intracellular mechanisms (and possibly autocrine signaling through chemerin_1_), β-catenin is targeted for phosphorylation and proteasomal degradation reducing the expression of TCF/LEF genes and inhibiting (−) cell growth, proliferation, and invasion. APC, APC Regulator of Wnt Signaling Pathway; GSK3β, glycogen synthase kinase 3β; CK-1, casein kinase 1. (?) unknown or possible but unconfirmed mechanism.

To evaluate the functional effects of chemerin in adrenocortical carcinoma, Li-Chittenden et al. performed a series of in vitro studies comparing the effects of transient human chemerin overexpression in H295R and SW13 adrenocortical carcinoma or HEK293 human embryonic kidney cells to exogenous chemerin treatment [79]. The effects of the transient transfection were cell-dependent and reduced the proliferation of the HEK293 cells and the cell invasion of the H295R cells but had no effect on proliferation or invasion of the SW13 cells. Furthermore, the transient transfection of the chemerin construct did not affect the migration of any of the cancer cell lines. Treatment with physiological levels of active chemerin had no impact on cell proliferation, invasion, or migration. The differential effects of chemerin overexpression versus exogenous treatment have also been observed with respect to adipocyte function [101]. While the exact mechanism is unknown, possibilities include differential post-translation processing of recombinant chemerin in a bacterial system versus in human cells, differential proteolytic processing of endogenous chemerin, or novel intracellular functions independent from chemerin_1_ and chemerin_2_ function. In support of the latter possibility, the cells tested in this study had barely detectable chemerin_1_ [79]. However, no assessment of chemerin_2_ levels was made. In further support of a direct tumor suppressive (rather than immune-mediated) effect of chemerin, H295R cells with stable expression of human chemerin had decreased colony formation and invasion in in vitro assays and formed smaller tumors when xenografted into the flanks immunodeficient T-cell deficient athymic nude and T, B, and NK-cell deficient and macrophage and dendritic cell-impaired NOD Scid γ mice. Further probing the tumor-suppressive mechanisms revealed that chemerin inhibits the Wnt/β catenin pathway, which is commonly activated in adrenocortical carcinoma and associated with higher tumor grades and decreased overall survival and disease-free survival (Figure 6) [102,103,104]. Thus, a reduction of chemerin in benign adrenal adenoma and adrenocortical carcinoma would be expected to lead to increased Wnt/β-catenin activity. Whether this plays a role in the initiation of adrenocortical carcinoma remains to be determined. The findings of Li-Chittenden et al. are consistent with previous studies in mesenchymal stem cells that showed chemerin_1_ is a Wnt responsive gene that functions as a negative feedback regulator of the Wnt/β-catenin signaling pathway [105]. Thus, it would be interesting to determine if the low chemerin_1_ expression is a factor that contributes to activation in Wnt/β-catenin activation in adrenocortical carcinoma. A second possible tumor-suppressive mechanism is through inhibition of p38 MAPK signaling. 

## 9. Renal Carcinoma 

**Figure 7:**
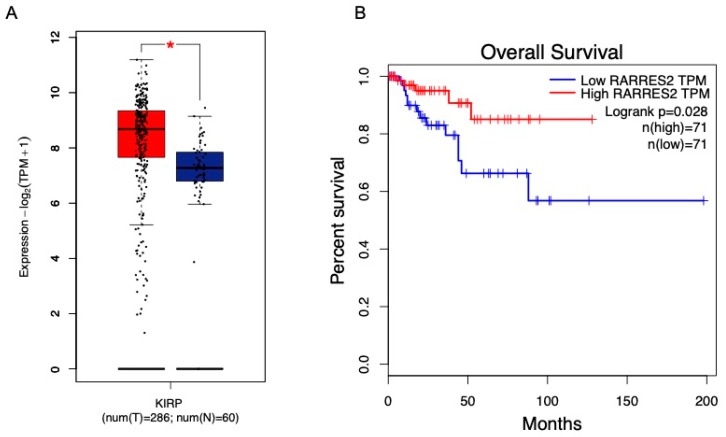
*Chemerin* expression is increased in papillary renal cell carcinoma (pRCC or KIRP) and is associated with higher overall survival. The Gene Expression Profiling Interactive Analysis (GEPIA) web server [106] was used for RNA sequencing expression analysis of *chemerin* in pRCC (red bar) and normal renal samples (blue bar) from the Cancer Genome Atlas (TCGA) and Genotype-Tissue Expression (GTEx) projects (**A**). The GEPIA web server survival analysis tool [106] was used to compare the overall survival of the quartile of pRCC patients with the highest *chemerin* expression (red line) versus the quartile of pRCC patients with the lowest *chemerin* expression (blue line) (**B**). * *p* < 0.01. TPM, transcripts per kilobase million.

An analysis of chemerin expression in RNA sequencing data available in the Cancer Genome Atlas (TCGA) and Genotype-Tissue Expression (GTEx) projects using the Gene Expression Profiling Interactive Analysis (GEPIA) web server revealed that papillary renal cell carcinoma (pRCC) has significantly upregulated *chemerin* expression (Figure 7a) [106]. This is opposite to the majority of tumors that display decreased *chemerin*. While there is little information regarding the potential impact of elevated chemerin expression in renal carcinoma, a recent study sheds some light on the matter [107]. pRCC accounts for approximately 20% of all renal cancers. A unique feature of pRCC is the focal aggregation of foam cell macrophages inside the papillae. In the study by Krawczyk et al., foamy macrophages were histologically identified in 82% of pRCC tumors and the macrophages expressed cell surface markers CD689 and CD163 that are characteristic of the M2 anti-inflammatory phenotype [107]. The researchers hypothesized that the pRCC cells must secrete factors that recruit monocytes and contribute to their differentiation into foamy macrophages. Utilizing freshly isolated primary pRCC cultures, the prototypical monocyte chemoattract proteins were not detected in conditioned media. Rather the most abundant secreted cytokines/chemokines were chemerin, interleukin-8 (IL-8), and CXCL16. Confirming their hypothesis, these cytokines, alone or in combination, stimulated the migration of human monocytes in transwell chemotaxis assays. Furthermore, conditioned pRCC medium shifted macrophages from an M1 to M2 phenotype and promoted their lipid accumulation. Thus, it is possible that elevated chemerin expression in pRCC could contribute to monocyte recruitment and differentiation into lipid-containing foam cells. However, the exact role chemerin on pRCC tumor biology and the tumor microenvironment is not known. A GEPIA survival analysis conducted with data from TCGA and GTEx indicated the quartile of patients with the highest tumor *chemerin* expression had better overall survival than those in the lowest quartile (Figure 7b), providing preliminary support that the differential *chemerin* expression could be functionally important in pRCC [106].

## 10. Thyroid Cancer

**Figure 8:**
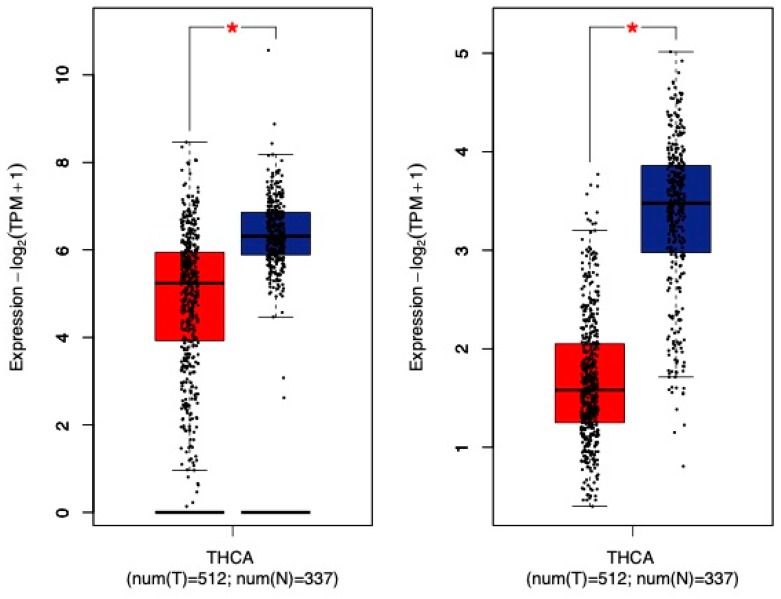
*Chemerin* and *chemerin_1_* are decreased in thyroid carcinoma (THCA). The Gene Expression Profiling Interactive Analysis (GEPIA) web server [106] was used for RNA sequencing expression analysis of *chemerin* and *chemerin_1_* in thyroid carcinoma (red bars) and normal renal samples (blue bars) from the Cancer Genome Atlas (TCGA) and Genotype-Tissue Expression (GTEx) projects. * *p* < 0.01. TPM, transcripts per kilobase million.

Thyroid carcinoma is the most common of the endocrine cancers, typically affects women more than men, and is most often observed in the fourth and fifth decades of life. Thyroid cancer is an obesity-associated cancer with increased risk with increasing BMI and weight gain [6,108]. The mechanisms linking obesity to thyroid cancer are not completely understood, but there has been considerable interest in the role of adipocytokines. Recently, Warakomski et al. sought to evaluate the relationship between serum chemerin, IL-6, leptin, and adiponectin and papillary thyroid cancer [66]. Overweight or obese patients (BMI > 25 kg/m^2^) did not have larger tumor sizes but were more often at an advanced clinical stage (II, III, or IV). While the overweight and obese subjects had higher preoperative serum chemerin (Table 1), there was no specific association between serum chemerin concentration and clinical stage. However, those subjects with higher leptin and IL-6 tended to have a more advanced clinical stage. While a direct association of chemerin with papillary thyroid cancer could not be determined in this study, there were a number of important limitations. First, the majority of study subjects (144) were diagnosed with stage I cancer, and thus, the sample size may have been too small for the advanced clinical stages to determine a relationship. Second, the study only evaluated serum chemerin concentration and did not perform any functional studies. GEPIA Analysis [106] of RNA sequencing data shows that *chemerin* and *chemerin_1_* are expressed in thyroid tissue and significantly downregulated in thyroid cancer samples (Figure 8). *Chemerin_2_* and *CCRL2* expression were lower and did not differ between tumor samples and normal thyroid tissue. It would be interesting for future studies to evaluate the relevance of *chemerin* and *chemerin_1_* downregulation to thyroid tumor biology and if chemerin signaling has direct effects on thyroid cancer cells.

## 11. Breast Cancer

Studies of the relevance of chemerin to breast cancer have provided conflicting results. As reviewed in greater detail by Treeck et al. [53], and originally reported by El-Sagheer et al. [109], chemerin protein expression was higher in cancerous versus adjacent healthy tissues and in metastatic lymph nodes compared to non-metastatic malignant tissues. Tumour chemerin expression was also negatively correlated with estrogen and progesterone receptor levels as well as five-year-disease-free survival rates [109]. In contrast, Pachynski et al. [110] reported that increased *chemerin* expression promoted the recruitment of immune effector cells to the tumor microenvironment and thus, initiated anti-cancer effects. An analysis of several breast cancer databases revealed that *chemerin* expression was significantly downregulated in malignant breast tissue compared to adjacent healthy tissue and that low *chemerin* expression was associated with poorer survival outcomes [110]. Consistent with this, quantitative real-time PCR and in situ hybridization demonstrated significantly lower *chemerin* expression in invasive/infiltrating ductal carcinoma and invasive/infiltrating lobular carcinoma tissues versus healthy breast tissue samples [110]. These studies demonstrate an interesting finding that while *chemerin* expression is downregulated in breast cancer tissues, protein expression is upregulated. These findings suggest the potential for translational and post-translational regulatory mechanisms in breast cancer cells, which differentially affect chemerin mRNA and protein expression. Further research is required to determine the cause of the inverse relationship between these expression levels.

Pachynski et al. [110] also examined the impact of chemerin expression levels in the EMT6 murine model of mammary carcinoma. While lentiviral-induced expression of chemerin did not impact cell proliferation in vitro, tumors generated from high chemerin-secreting (HCS) EMT6 clones exhibited significantly lower growth compared to those derived from low chemerin-secreting (LCS) secreting or control EMT6 cells in an in vivo xenograft model [110]. Furthermore, there was a significant increase in the relative proportions of T cells, CD4+ T cells, and NK cells in the HCS-EMT6 tumors compared to controls, and this was associated with tumor suppression [110]. Depletion experiments indicated a critical role of NK cells and CD8+ T cells in the tumor suppression response to chemerin, while the depletion of CD4+ T regulatory cells enhanced tumor suppression [110]. Thus, a plausible mechanism by which chemerin may affect breast cancer progression is via the recruitment of immune cells to the tumor microenvironment. In contrast to the findings of Pachynski et al. [110], El-Sagheer et al. [109] suggested a potential for pro-tumorigenic effects via the influence on the breast cancer stem cell (BCSC) phenotype. It is well established that inflammatory cytokines can promote epithelial–to–mesenchymal transformation and angiogenesis, among other pro-cancer effects [111,112]. Notably, IL-6, a pro-inflammatory cytokine secreted by several immune cell types, has been shown to play a role in inducing the de-differentiation of malignant cells to BCSCs [111,113]. Although research is limited, it is believed that BCSCs contribute to tumor progression and poor prognosis in breast cancer patients [112,114]. The possibility that chemerin-mediated recruitment of immune effectors to the tumor microenvironment contributes to poor prognosis via the promotion of BCSC phenotype is an intriguing possibility that remains to be experimentally tested.

Akin et al. [67] reported correlations between serum chemerin concentrations and several clinical factors such as diabetes, age of diagnosis, BMI, hypertension, and menopause, but found no significant difference between serum chemerin levels in breast cancer patients with metastatic and non-metastatic cancer (Table 1). While these findings suggest that serum chemerin is not associated with breast cancer stage, an important limitation of this study was the lack of a control group without breast cancer. Thus, further studies are needed to determine if there is a relationship between chemerin levels and breast cancer, per se.

As reviewed in greater detail by Treeck et al. [53], and originally reported by Sarmadi et al., expression of the atypical chemokine receptor, CCRL2, has been observed in human malignant breast tissues samples, but not in adjacent non-cancerous tissues and exhibited no significant association with stage [115]. It has been hypothesized that due to the ability of CCRL2 to sequester chemerin and thereby limit its ability to act on signaling receptors, the upregulation of CCRL2 in malignant breast tissues may function as an immune evasion mechanism [115]. However, this idea conflicts with observations in hepatocellular carcinoma, where chemerin_1_ and CCRL2 appear to act cooperatively in inhibiting infiltration of MDSCs into the tumor microenvironment [92].

## 12. Ovarian Cancer 

In the seminal study that identified chemerin as a ligand for chemerin_1,_ chemerin was found to be abundant in ascitic fluid of ovarian cancer patients [42]. The authors suggested that chemerin signaling through chemerin_1_ could be involved in diseases with a strong inflammatory component, such as autoimmune disorders and cancer [42]. While this study provided the first suggestion that chemerin could be involved in ovarian cancer, research in this area is very limited. As reviewed in greater detail by Treeck et al. [53], and reported originally by Hoffman et al. [116] and Reverchon et al. [117], experimental evidence exists for differential expression of chemerin and the cognate receptors, as well as the biological impact of this signaling pathway in several ovary cell types (normal and cancerous). However, further research is necessary to determine the impact on ovarian cancer development, progression, and the efficacy of hormonal therapies. 

## 13. Central Nervous System Cancers

At present, investigation of the relevance of chemerin and the cognate receptors to cancers of the nervous system is very limited. Tummler et al. [118] reported that expression of chemerin_1_, but not chemerin_2_, was elevated (versus neural crest and benign neurofibroma cells) in tumors from patients with neuroblastoma, a pediatric cancer of the peripheral nervous system. Moreover, a significant correlation was found between high expression of chemerin_1_, chemerin_2_, or CCRL2 and a decrease in overall survival probability. Exogenous chemerin stimulated MAPK and Akt phosphorylation, increased calcium mobilization and MMP-2 secretion from neuroblastoma SK-N-AS cells, while treatment with the putative chemerin_1_ inhibitor α-NETA reduced the viability and clonogenicity of these cells. Consistent with the latter, α-NETA impaired tumor growth in vivo in a murine SK-N-AS xenograft model. Taken together, these data provide evidence that chemerin/CMKLR1 signaling promotes neuroblastoma development through direct effects on tumor cells and the tumor microenvironment.

Zhao [46] reported that while the relatively inactive chemerin isoform chemerin163 is the major contributor (~80%) to total plasma chemerin, the majority (~55%) of cerebrospinal fluid chemerin is comprised of the bioactive isoforms chemerin158 and chemerin157. Silico analysis of published microarray datasets indicated that chemerin, but not *chemerin_1_* or *CCRL2* mRNA levels were elevated in grade III and IV (malignant) tumors compared with grade II glioma [46]. Furthermore, treatment of human U-87 MG glioblastoma cells with chemerin157 triggered a dose-dependent transient increase of intracellular calcium levels. Taken together, these data reinforce the concept that anatomical locations can differ with respect to the spectrum of chemerin isoforms and indicate that glioblastoma cells both secrete and respond to chemerin. However, it is important to note that chemerin has not been linked to glioblastoma outcomes nor to biological effects that directly or indirectly promote the malignancy of glioblastoma cells.

## 14. Lung Cancer

Much of the research into the role of chemerin in lung cancer stems from clinical studies of patients with non-small cell lung carcinoma (NSCLC). Several clinical studies have reported that patients with lung cancer had higher circulating chemerin concentrations than controls and/or that serum chemerin concentrations were positively associated with several clinical parameters including stage, lymph node infiltration, and distant metastasis (Table 1) [68,69,70,119]. While higher serum chemerin concentrations are generally associated with pro-cancer effects in NSCLC, many findings point to a role of localized chemerin in promoting anti-cancer effects via the recruitment of NK cells to the tumor microenvironment [120,121]. Thus, a downregulation of chemerin secretion by tumor cells may promote immune evasion and consequently, poor clinical outcomes. Further empirical research is required to fill in the current gaps in the literature with respect to the causal effect of chemerin on lung cancer development and progression, as well as its effects on biological indicators of cancer such as proliferation, metastasis, and invasion. We refer the interested reader to the article by Treeck et al. [53] in this issue that provides a more complete assessment of chemerin in NSCLC.

## 15. Pancreatic Cancer

Patients that are positive for pancreatic ductal adenocarcinoma exhibit significantly higher plasma chemerin concentration than healthy volunteers (Table 1) [71]. Despite this marked difference between pancreatic cancer patients and healthy controls, this study found no significant correlation between cancer stage and plasma concentration of chemerin, nor any correlation between chemerin concentration and resectable versus unresectable tumors [71]. The authors proposed that chemerin concentration could be used as a biomarker for the presence of cancer, where a plasma concentration of >219.67 ng/mL showed 80% sensitivity and 83% specificity for the presence of disease [71]. 

## 16. Prostate Cancer

No significant difference in serum chemerin concentration was found between patients with prostate cancer and those with benign prostatic hyperplasia, however, differences were identified between cancer patients with different Gleason scores, a progressive measurement of prostate cancer aggressiveness as determined by tumor cell differentiation [72]. Serum chemerin concentration was observed to increase with Gleason score, where tumors with a score of ≥8, 7, and ≤6 were significantly different from one another [72]. There was also a positive correlation between the serum levels of chemerin and IL-6 [72]. Comparing non-obese to obese patients with prostate cancer who subsequently underwent radical prostatectomy, there was no significant difference found in serum chemerin concentration based on BMI prior to surgery (Table 1) [73]. Furthermore, serum chemerin was not found to be a predictive factor for advanced tumor stage in the overall population nor in patients with a BMI of > 25 kg/m^2^ [73]. These latter findings argue against a role of adipose-derived chemerin in prostate cancer. However, while serum chemerin concentrations increased with Gleason score, the opposite effect was observed for *chemerin* expression in prostate tumor tissue [122]. Furthermore, *chemerin* was downregulated in prostate cancer as compared to benign prostate tissues, with greater downregulation observed in castration-resistant prostate cancers [123]. While chemerin_1_ and chemerin_2_ expression were not evaluated, CCRL2 mRNA and protein levels were reported to be increased in prostate cancer PC3 cells, and *CCRL2* expression increased in prostate cancer tissues versus prostate tissues from patients with benign prostatic hyperplasia [124]. However, the impact of these changes on chemerin signaling in tumor cells or the tumor microenvironment has not been evaluated.

## 17. Conclusions

Obesity is a major global health concern that has been linked to the development of many prevalent metabolic disorders such as type 2 diabetes, hyperlipidemia, and cardiovascular disease. There is also an increasing awareness that obesity represents a significant risk factor for the development of several malignancies. While our current understanding of the pathophysiological mechanisms linking obesity to cancer is evolving, growing interest has focused on the role of adipocyte-secreted signaling molecules as key mediators linking these disorders. Among these, circulating levels of the adipokine chemerin are well established to be directly related to adipose tissue mass and have been implicated in several obesity-related metabolic comorbidities. Altered levels of chemerin and the cognate receptors, chemerin_1_, chemerin_2_, and CCRL2 have also been identified in several cancer types and many of the fundamental biological activities (e.g., chemotaxis, proliferation, differentiation) of chemerin have the potential to affect tumorigenesis and tumor progression. These effects may be elicited through immune-independent mechanisms that directly impact the growth and tumorigenicity of cancer cells and/or immune-dependent effects that influence the composition of the tumor microenvironment.

At present, epidemiological studies have introduced the potential utility of this adipokine as a potential biomarker for several malignancies, and clinical and empirical evidence supports both pro- and anti-cancer effects of chemerin. This suggests that the biological actions of chemerin with respect to cancer are highly contextual and dependent upon a number of factors that are important areas of further investigation. A fundamental issue in this regard is the large discrepancy (up to three orders of magnitude; see Table 1) in the reported values of serum/plasma chemerin concentration in the clinical literature—even among control populations. While this may reflect the inherent heterogeneity of the control populations, assay-dependent factors may also play a role. It is critical that methodologies are both reported in appropriate detail and rigorously validated with respect to sensitivity and specificity. Moreover, the overwhelming majority of studies have utilized methodologies that are unable to distinguish between chemerin isoforms and/or only test the actions of chemerin157. It will be important going forward to consider the actions of other known isoforms of chemerin as their relative abundance may differ depending upon anatomical location and their biological actions may be cell- and tissue-dependent. Similarly, most research to date has focused on chemerin_1_-dependent actions of chemerin. Elucidation of the role of chemerin_2_ and CCRL2 and the chemerin isoform-selectivity of these receptors in the context of cancer are priority areas for investigation. Moreover, while there has been considerable interest in the relationship of systemic concentrations of adipose-derived chemerin to cancer development and prognosis, comparatively little attention has been applied to the relevance of locally-derived chemerin secreted from cells located in the affected tissue or tumor microenvironment. This may be of particular importance to malignancies such as breast cancer where adipocytes are commonly found in close proximity to tumors and where evidence exists for an influence on tumor development and progression. Finally, most research regarding the impact of adipokines on cancer has focused on a single molecule. It is well known that the relative amounts and spectrum of adipokines is affected by adiposity and adipocyte function. Hence, while challenging, it will be important to apply a more holistic experimental approach to consider the interactions of multiple adipokines and consider synergistic and/or antagonistic effects in different tumor types and at different stages of tumor development.

## Figures and Tables

**Table 1:** Summary of chemerin concentrations and tissue expression data.

Cancer Type	Demographics				Serum, Plasma, or Tissue Chemerin in ng/mL
	Group, *n*	Age in Years	Sex	BMI kg/m^2^	
OSCC [54]					serum
	OSCC, 15	47.7 ± 14.1	M6/F9	22.8 ± 1.1	655 ± 150 ^†^
	OPML, 15	42.3 ± 11.0	M5/F10	22.4 ± 1.1	408 ± 85 *
	Controls, 15	43.3 ± 11.8	M7/F8	22.7 ± 1.5	187 ± 13
					salivary fluid
	OSCC, 15	47.7 ± 14.1	M6/F9	22.8 ± 1.1	13.2 ± 3.8 ^†^
	OPML, 15	42.3 ± 11.0	M5/F10	22.4 ± 1.1	9.1 ± 1.9 *
	Controls, 15	43.3 ± 11.8	M7/F8	22.7 ± 1.5	3.1 ± 0.7
Colorectal [59]					Serum
	Patients, 41	55 (32–75)	M28/F13	25.8 (16.2–35.5)	390 (250–630)
	Controls, 27	43 (18–64)	M15/F12	26.6 (21.5–45.8)	340 (270–480)
Colorectal [60]					plasma
	Patients, 221	50 ± 9	62.1% F	16.5% > 30	148 (50–370)
Gastric [61]					plasma
	Patients, 196	44.4% ≥ 60	M112/F84	23.0 ± 3.1	53.1 ± 19.0 *
	Controls, 196	55.6% < 60	Matched	23.4 ± 3.5	31.3 ± 11.3
Colorectal [62]					serum
	Patients, 32	57.6 ± 6.5	M22/F10	25.8 ± 4.2	377.0 ± 80 *
	Controls, 20	58.4 ± 7.2	M14/F6	26.7 ± 5.3	87.8 ± 22.0
Colorectal [63]					serum
	Survivors, 110	56.3 ± 9.3	M55/F55	23.3 ± 3.1	105 ± 14
Gastric [64]					serum
	Patients, 36	47–83	M19/F17		42 *
	Controls, 40	31–68	M27/F13	non-obese	28
HCC [65]					serum
	Patients, 44	71 (50–82)	M29/F15	22.5 (15.6–33.5)	130 (80–312)
Thyroid [66]					serum
	BMI < 25, 51	41.2 ± 11.9	F51	21.8 ± 2.1	212 ± 47
	BMI ≥ 25, 126	55.4 ± 12.7	M26/F100	30.7 ± 4.1	229 ± 50 *
Breast [67]					serum
	Metastatic, 37	52.3 ± 11.8	F37	29.1 ± 5.5	250 ± 59
	Non-Met, 80	51.7 ± 12.5	F80	28.6 ± 4.9	261 ± 73
	All, 117	51.9 ± 12.2	F117	28.7 ± 5.1	257 ± 69
CNS [46]	GBM, 12	N/A	N/A	N/A	CSF
					chem157S‡—0.2 ± 0.3
					chem158K‡—5.1 ± 3.9
					chem163S‡—3.0 ± 2.4
	ODC, 12	N/A	N/A	N/A	chem157S—0.7 ± 1.3
					chem158K—3.8 ± 3.8
					chem163S—2.9 ± 2.5
	NC CNS, 7	N/A	N/A	N/A	chem157S—1.0 ± 0.8
					chem158K—6.3 ± 4.8
					chem163S—5.5 ± 3.8
	Controls, 9	N/A	N/A	N/A	plasma
					chem157S—0.7 ± 0.8
					chem158K—8.1 ± 2.9
					chem163S—40 ± 7.9
NSCLC [68]					serum
	Patients, 110	65.1	M91/F19	26.4	245 *
	Controls, 110	65.0	M91/F19	27.7	203
NSCLC [69]					serum
	Patients, 189	61.8 ± 11.2	M124/F65	NA	1.78 ± 0.57 *
	Controls, 120	62.6 ± 8.9	M69/F51		1.20 ± 0.23
Lung [70]					plasma
	Patients, 42	56 (44–78)	M26/F16	N/A	1.97 ± 0.37 *
	Controls, 31	48 (32–64)	M18/F13		1.11 ± 0.25
Pancreatic ductal [71]					serum
	Patients, 25	63.0 ± 9.8		24.5 (21.7–27.8)	272 (221–314) *
	Controls, 36	37.6 ± 6.4	M36	26.1 (24.2–29.5)	193 (173–214)
Prostate [72]					serum
	All patients, 74	67.1 ± 8.5	M74	27.9 ± 3.3	273 ± 29
	BPH, 66	61.5 ± 10.3	M66	27.3 ± 4.0	268 ± 83
	WD, 24	64.6 ± 8.5	M24	27.2 ± 3.6	237 ± 72 ^+^
	MD, 28	66.7 ± 8.8	M28	28.0 ± 2.8	274 ± 60 ^+^
	PD, 22	70.2 ± 7.5	M22	28.3 ± 3.4	313 ± 93 ^+^
Prostate [73]					serum
	Non-obese, 25	68 (64–73)	M25	23.0 (21.5–24.3)	74.0 (59.4–88.1)
	Obese, 37	64 (60–67)	M37	26.7 (25.7–27.6)	75.0 (65.6–82.3)

Parentheses indicate the range of reported values. OSCC, oral squamous cell carcinoma; OPML, oral premalignant lesion; HCC, hepatocellular carcinoma; NSCLC, non-small cell lung cancer; GBM, malignant glioblastoma; ODC, oligodendrocytoma; NC CNS, non-cancer CNS disease; BPH, benign prostatic hyperplasia; WD, well differentiated prostate cancer (Gleason score ≤ 6); MD, moderately differentiated (Gleason 7); PD, poorly differentiated (Gleason ≥ 8). ‡ Number refers to the number of amino acids in the processed chemerin protein, ^†^ Significant compared to the other two groups; * significant compared to control group; ^+^ significant compared to other Gleason scores.

## Abbreviations

AKT	protein kinase B
APC	APC regulator of Wnt signaling pathway
ATM	adjacent tissue myofibroblast
BCSC	breast cancer stem cell
BE	Barrett’s esophagus
BPH	benign prostatic hyperplasia
CA125	cancer antigen 125
CA 15-3	cancer antigen 15-3
CAM	cancer associated myofibroblast
CCRL2	C-C Chemokine Receptor-Like 2
CEA	carcinoembryonic antigen
chemerin_1_	chemerin receptor 1
chemerin_2_	chemerin receptor 2
CK-1	casein kinase 1
CMKLR1	Chemokine-Like Receptor 1
CRP	C-reactive peptide
CYFRA 21-1	cytokeratin 19 fragment 21-1
ECM	extracellular matrix
EMT	epithelial-to-mesenchymal transformation
ERK	extracellular-related kinase
GBM	malignant glioblastoma
GEPIA	Gene Expression Profiling Interactive Analysis
GM-CSF	granulocyte-macrophage colony-stimulating factor
GPR1	G Protein-coupled Receptor 1
GSK3β	glycogen synthase kinase 3β
GTEx	Genotype-Tissue Expression
HCC	hepatocellular carcinoma
HCS	high chemerin-secreting
hGC	human granulosa cells
IFN-I	type 1 interferon
IFNγ	interferon gamma
IFNγT	interferon γ-secreting T cells
IL-6	interleukin-6
IL-8	interleukin-8
JNK	c-jUN N-terminal kinase
KGN	human ovarian granulosa-like tumor
LCS	low chemerin-secreting
MAPK	mitogen-activated protein kinase
MD	moderately differentiated prostate cancer (Gleason 7)
mDC	myeloid dendritic cell
MDSC	myeloid-derived suppressor cells
MIF	macrophage inhibitory factor
MIP3α	macrophage inflammatory protein-3 alpha
MMP	matrix metalloproteinase
MSC	mesenchymal stromal cell
NASH	non-alcoholic steatohepatitis
NASH-HCC	non-alcoholic steatohepatitis with dimethylnitrosamine-induced hepatocarcinoma
NC CNS	non-cancer CNS disease
NF-κB	nuclear factor kappa B
NK	natural killer
NSCLC	non-small cell lung cancer
NTM	normal tissue myofibroblast
ODC	oligodendrocytoma
OPL	oral pre-malignant lesion
OSC	oesophageal squamous cancer
OSCC	oral squamous cell carcinoma
pRCC	papillary renal cell carcinoma
PD	poorly differentiated prostate cancer (Gleason ≥8)
pDC	plasmacytoid dendritic cell
PI3K	phosphoinositide 3-kinase
PKC	protein kinase C
PTEN	phosphatase and tensin homolog
PVTT-1	portal vein tumor thrombus cells
PVTT-1-Che	chemerin-overexpressing portal vein tumor thrombus cells
SCCOT	squamous cell carcinoma of the oral tongue
TAM	tumor associated macrophage
TCGA	The Cancer Genome Atlas
TIMP-1	tissue inhibitor of metalloproteinase 1
TIMP-2	tissue inhibitor of metalloproteinase 2
TME	tumor microenvironment
TNM	tumor-node-metastasis
TPM	transcripts per kilobase million
Treg	regulatory T cell
VEGF	vascular endothelial growth factor
WD	well differentiated prostate cancer (Gleason score ≤ 6)
